# High proportion of healthcare-associated urinary tract infection in the absence of prior exposure to urinary catheter: a cross-sectional study

**DOI:** 10.1186/2047-2994-2-5

**Published:** 2013-02-07

**Authors:** Ilker Uçkay, Hugo Sax, Angèle Gayet-Ageron, Christian Ruef, Kathrin Mühlemann, Nicolas Troillet, Christiane Petignat, Enos Bernasconi, Carlo Balmelli, Andreas Widmer, Karim Boubaker, Didier Pittet

**Affiliations:** 1 Infection Control Programme and WHO Collaborating Centre on Patient Safety, University of Geneva Hospitals and Faculty of Medicine, 4 Rue Gabrielle Perret-Gentil, 1211, Geneva 14, Switzerland; 2 Orthopaedic Surgery Department, University of Geneva Hospitals and Faculty of Medicine, Geneva, Switzerland; 3 Department of Infectious Diseases, University of Geneva Hospitals and Faculty of Medicine, Geneva, Switzerland; 4 Division of Infectious Diseases, University Hospital of Zurich, Zurich, Switzerland; 5 Department of Infectious Diseases, University Hospital Bern, Bern, Switzerland; 6 Department of Infectious Diseases, Central Institute of the Valais Hospitals, Sion, Switzerland; 7 Department of Hospital Preventive Medicine, Centre Hospitalier Universitaire Vaudois and University of Lausanne, Lausanne, Switzerland; 8 Department of Infectious Diseases, Ospedale Civico, Lugano, Switzerland; 9 Division of Infectious Diseases, University of Basel Hospitals, Basel, Switzerland; 10 Swiss Federal Office of Public Health, Bern, Switzerland; 11 Current addresses: Department of Infectious Diseases and Hospital Hygiene, University of Zurich, Zurich, Switzerland; 12 Current addresses: Hirslanden Clinics, Zurich, Switzerland; 13 Current addresses: Public Health Service for the Canton of Vaud, Lausanne, Switzerland

**Keywords:** Prevalence, Urinary catheter, Acute care, Urinary tract infection, Nosocomial, Risk factors

## Abstract

**Background:**

Exposure to urinary catheters is considered the most important risk factor for healthcare-associated urinary tract infection (UTI) and is associated with significant morbidity and substantial extra-costs. In this study, we assessed the impact of urinary catheterisation (UC) on symptomatic healthcare-associated UTI among hospitalized patients.

**Methods:**

A nationwide period prevalence survey of healthcare-associated infections was conducted during 1 May to 30 June 2004 in 49 Swiss hospitals and included 8169 adult patients (4313 female; 52.8%) hospitalised in medical, surgical, intermediate, and intensive care wards. Additional data were collected on exposure to UC to investigate factors associated with UTI among hospitalised adult patients exposed and non-exposed to UC.

**Results:**

1917 (23.5%) patients were exposed to UC within the week prior to survey day; 126 (126/8169; 1.5%) developed UTI. Exposure to UC preceded UTI only in 73 cases (58%). By multivariate logistic regression analysis, UTI was independently associated with exposure to UC (odds ratio [OR], 3.9 [95% CI, 2.6-5.9]), female gender (OR, 2.1 [95% CI, 1.4-3.1]), an American Society of Anesthesiologists’ score > 2 points (OR, 3.2 [95% CI, 1.1-9.4], and prolonged hospital stay >20 days (OR, 1.9 [95% CI, 1.4-3.2]. Further analysis showed that the only significant factor for UTI with exposure to UC use was prolonged hospital stay >40 days (OR, 2.9 [95% CI, 1.3-6.1], while female gender only showed a tendency (OR, 1.6 [95% CI, 1.0-2.7]. In the absence of exposure to UC, the only significant risk factor for UTI was female gender (OR, 3.3 [95% CI, 1.7-6.5]).

**Conclusions:**

Exposure to UC was the most important risk factor for symptomatic healthcare-associated UTI, but only concerned about half of all patients with UTI. Further investigation is warranted to improve overall infection control strategies for UTI.

## Background

Indwelling urinary catheters (UC) are an integral part of medicine today [1,2], and as many as one-quarter of all patients require their placement during hospital stay [3]. Exposure to UC is currently considered the most important risk factor for healthcare-associated urinary tract infection (UTI) and is associated with significant morbidity [3-5] and substantial extra-costs [6,7]. The literature suggests that the rate of UTI acquisition is 5% per day of UC use [3]. Despite the harm potential and the existence of educational programmes to prevent unnecessary catheterisation, UC continues to be frequently used or maintained without clear indications [3,4,8].

In Switzerland, nationwide period prevalence surveys of healthcare-associated infections have been regularly conducted for the past 15 years and provide a unique opportunity to gain insight into their epidemiology [9-12]. During the 2004 survey, we collected additional data on exposure to UC and evaluated factors associated with UTI among hospitalised adult patients exposed and non-exposed to UC.

## Methods

### Setting and study organization

Five nationwide period prevalence surveys of healthcare-associated infections have been conducted since 1996 in Switzerland in over 100 acute care hospitals [9-12]. These were coordinated by *SwissNOSO*, an independent panel of experts in infectious diseases and hospital epidemiology representing all Swiss university-affiliated hospitals. Hospital participation was voluntary. Observers were infection control practitioners who attended at least three one-day training sessions during which detailed documentation was provided in their native language, including the study protocol, standardised case report form, written definitions for all study variables, practical exercises, and code lists [9]. The prevalence study was scheduled to assess all healthcare-associated infections. All nurses and physicians performing the study were trained to distinguish between community and healthcare-associated infections; the latter was defined as symptomatic infection occurring after 48 h of hospital admission. Infection control practitioners from centres with and without experience in prevalence studies were grouped as study teams and supervised by an infection control physician at each centre [9,12].

### Study population and methodology

All patients hospitalised for at least 24 h during the survey conducted in May-June 2004 were assessed using a standardised case report form. Dermatological, ophthalmological, paediatric, and psychiatric wards were excluded, as well as long-term care sectors, defined as wards with a median hospital stay >30 days. In each hospital, one or several teams of two trained observers with experience in infection control reviewed simultaneously all medical and nursing charts and evaluated patients for the presence of healthcare-associated infections and potential risk factors within a fixed time window, defined as the survey day plus the six previous days. Observers rated their interpretation of chart data on a six-point Likert scale (1 = strongly disagree; 6 = strongly agree). All results were checked by the data manager and physicians in the study centre for plausibility and completeness, and corrected if necessary. The following variables were assessed for each patient: gender; age; immune suppression; hemiplegia; surgery; antibiotic use; hospital size; ward type; and length of hospital stay at time of survey. All non-surgical wards, e.g., neurology, rheumatology, were classified as medical wards. Acuity and severity of the underlying illness were assessed by the American Association of Anesthesiologists’ (ASA) score [13], McCabe and Jackson classification [14], Charlson comorbidity index [15], and nursing workload on survey day [16].

### Definitions

Definitions for healthcare-associated infections were adapted from those of the United States Centers for Disease Control and Prevention (CDC) [17] as previously described [9,11]. UTI was the primary outcome and defined as an infection occurring after 48 h of hospitalisation, unless the patient was transferred from another hospital and cumulative hospital stay reached 48 h. Clinical (dysuria, pollakisuria, fever or shivering), laboratory (urine sedimentation profiles, urinary tract cultures), medical and/or radiological data (ultrasound or computed tomography scans) were required for the confirmation of infection in the absence of other signs or explications. Only the first episode of symptomatic UTI for each patient was recorded. Asymptomatic urinary tract colonization (CDC code, UTI-ASB) was not considered as UTI [9,10]. Previous exposure to UC was the secondary outcome. Exposure to UC, intensive care, and antibiotics were only considered when they lasted for at least 24 h. A condom collection system was not considered as UC. Duration of exposure to UC was censored at 30 days. Surgery was defined as any procedure performed in the operating room within 30 days prior to survey day.

### Statistical analysis

The primary outcome was the occurrence of symptomatic UTI. First, we compared patients with and without UTI by using Chi-2 or Fisher’s exact tests for categorical variables. We used Student’s *t*-test or the non-parametric Mann–Whitney test to compare continuous variables. To take into account hospital clustering, we used a logistic regression model with a random effect at the hospital level (cluster effect) to assess the independent association between the main predictor (previous exposure to UC) and UTI after adjustment for the main confounders (gender, age, severity scores [McCabe, ASA], length of hospital stay, co-morbidities). We used the same regression model to assess the independent association between the secondary outcome (use of UC) and various risk factors. For both models, we used a backward stepwise procedure by selecting all variables associated with a p <0.20 at univariate and kept in the model all those significantly associated with the outcome (p <0.05). As we suspected that the pathophysiology of UTI differed according to gender and those exposed/not exposed to UC, we tested for an interaction between both variables and provided a stratified model on urinary catheter exposure/gender. We tested also for all interactions that were biologically plausible. Finally, we assessed the independent association between prior exposure to UC (secondary outcome) and the main confounders (gender, age, severity scores [McCabe, ASA], hospital length of stay, co-morbidities). The significance level was 0.05 (two-tailed). Statistical testing was performed, using SAS statistical software, version 9.2 (SAS Institute).

## Results

A total of 8169 patients from 49 hospitals participated in the survey, representing a nationwide estimate of at least 30% of all hospitalised patients; 4313 (52.8%) were female [12]. Among these, 1917 (23.5%) were exposed to UC with an overall median duration of use of 4 days (interquartile range, 2–9 days). Of 126 patients overall who developed UTI (1.5%; 85 female; median age, 77 years), 73 (58%) had been exposed to UC within the week preceding UTI onset, while 53 (42%) had no exposure. Sixty-two percent of all case report forms for UTI patients were maximally rated (6/6 points on the Likert scale) by observers and a further 25% with almost complete agreement (5/6 points). Females were at higher risk for UTI (odds ratio [OR], 2.1 [95% confidence interval (CI), 1.4-3.1]). Four UTI episodes were classified as upper UTI or abscesses, and six were bacteraemic. We identified a total of 14 different causative pathogens, of which the most frequent in descending order were *Escherichia coli*, *Proteus* spp, *Klebsiella* spp, *Enterobacter* spp, and *Enterococcus* spp. On average, UTI was diagnosed 16 days after admission (range, 2 to 124 d). Patient population characteristics stratified according to the occurrence of UTI are shown in Table 1. The 38 episodes of asymptomatic urinary tract bacterial colonization were excluded according to our study protocol.

**Table 1:** **Patient characteristics according to the presence of healthcare-associated urinary tract infection (UTI) (n = 8169), *Swiss-NOSO *Nationwide Prevalence Study, 2004**

	**UTI**	**No UTI**	**P value**
	**(n = 126)**	**(n = 8043)**	
Urinary catheter exposure (%)			**<0.001**
Yes	73 (57.9)	1912 (23.8)	
No	53 (42.1)	6131 (76.2)	
Gender, female (%)	85 (67.5)	4228 (52.6)	**0.001**
Mean age (±SD)	71.8 (15.6)	62.4 (19.0)	**<0.001**
Age groups (%)			**<0.001**
<40 years	7 (5.7)	1395 (17.9)	
41-70 years	37 (30.3)	3208 (41.2)	
> = 71 years	78 (63.9)	3184 (40.9)	
Hospital size			0.09
<200 acute care beds	23 (18.3)	1994 (24.8)	
200-500 beds	40 (31.8)	2748 (34.2)	
> = 501 beds	63 (50.0)	3301 (41.0)	
Mean length of stay (±SD)	20.5(22.2)	11.4 (41.0)	<0.001*
Length of stay			**<0.001**
<20 days	89 (70.6)	6990 (86.9)	
21-40 days	22 (17.5)	677 (8.4)	
> = 41 days	15 (11.9)	374 (4.7)	
Recent stay in intensive care (%)	34 (27.0)	1030 (12.8)	**<0.001**
Hospitalisation ward (%)			0.003*
Medical ward	45 (35.7)	3192 (39.7)	
Surgical ward	50 (39.7)	3219 (40.0)	
Gynaecology/obstetrics	5 (4.0)	778 (9.7)	
Intensive care unit	7 (5.6)	285 (3.5)	
Medico-surgical	19 (15.1)	569 (7.1)	
Recent surgery (%)	61 (48.4)	3292 (40.9)	0.09
Mean ASA score (±SD)	2.81 (0.67)	2.33 (0.89)	<0.001*
ASA (%)			<0.001*
1 pt	4 (3.2)	1587 (19.8)	
2-3 pts	109 (87.2)	5802 (72.3)	
4-5 pts	12 (9.6)	634 (7.9)	
McCabe/Jackson (%)			0.01*
Non fatal	82 (65.1)	6143 (76.4)	
Fatal within 5 years	36 (28.6)	1455 (18.1)	
Fatal within 6 months	8 (6.4)	445 (5.5)	
Mean nursing workload (±SD)	204.0 (161.4)	168.3 (162.8)	0.008*
Mean Charlson index (±SD)	1.8 (2.1)	1.2 (1.8)	<0.001*
Charlson group (%)			**0.02**
0-3 pts	106 (84.1)	7267 (90.4)	
> = 4 pts	20 (15.9)	776 (9.7)	
Co-morbidities (%)			
Diabetes mellitus	19 (15.1)	1074 (13.4)	0.57
Immune suppression°	26 (20.6)	1047 (13.0)	**0.01**
Hemiplegia	24 (19.1)	777 (9.7)	**<0.001**
Dementia	9 (7.1)	290 (3.6)	**0.04**

*ASA*, American Society of Anesthesiologists; (%) = proportion of all surveyed patients according to number of the variable of interest among the study population; *SD* = standard deviation; °autoimmune disease, transplantation, hepatopathy, nephropathy, neoplasia.

### Multivariate adjustment

#### Overall UTI

By multivariate logistic regression analysis with a random effect at the hospital level, independent factors associated with higher odds for UTI were prior exposure to UC (odds ratio, OR, 3.9 [95% CI, 2.6-5.9]), female gender (OR, 2.1 [95% CI, 1.4-3.1]), ASA score of > 2 points (OR, 3.2 [95% CI, 1.1-9.4], and prolonged hospital stay > 20 days (OR, 1.9 [95% CI, 1.4-3.2]. When female gender and prior exposure were combined with UC as one risk factor, the likelihood of UTI increased by 10.4-fold compared to the combination of male gender and no prior exposure to UC. Women not exposed to UC had a 3.4-fold risk of UTI compared to men not exposed to UC. The likelihood of UTI increased independently with age, ASA group, and the length of hospital stay, after adjustment on the main other confounders (Tables 2 &3).

**Table 2:** **Multivariate logistic regression models clustered on hospitals presenting independent factors associated with urinary tract infection stratified on prior exposure to urinary catheter, *Swiss-NOSO *Nationwide Prevalence Study, 2004**

**Variables**	**Prior exposure to urinary catheter**			**No prior exposure to urinary catheter**		
	**(n = 1917)**			**(n = 5957)**		
	**Odds ratio**	**95% CI**	**P value**	**OR**	**95% CI**	**P value**
Age groups (%)			0.62			0.03*
<40 years	1.00	-	-	1.00	-	-
41-70 years	1.77	0.51-6.10	0.36	1.03	0.31-3.45	0.96
> = 71 years	1.84	0.54-6.26	0.33	2.53	0.81-7.94	0.11
Recent surgical intervention			0.75			0.59
No	1.00	-		1.00	-	
Yes	0.92	0.54-1.56		0.84	0.44-1.59	
ASA score (%)			0.09*			0.39
1 pt	1.00	-	-	1.00	-	-
2-3 pts	7.21	0.96-54.35	0.06	1.99	0.54-7.28	0.30
4-5 pts	4.52	0.51-39.74	0.17	1.11	0.18-6.78	0.91
Charlson group			0.55			0.76
0-3 pts	1.00	-		1.00	-	
> = 4 pts	1.30	0.55-3.08		0.85	0.30-2.40	
Length of stay			0.01*			0.09*
<20 days	1.00	-	-	1.00	-	-
21-40 days	1.88	0.92-3.85	0.08	2.01	0.95-4.22	0.07
> = 41 days	2.87	1.34-6.11	0.006	2.12	0.84-5.35	0.11
Recent stay in intensive care			0.99			0.05*
No	1.00	-		1.00	-	
Yes	1.00	0.58-1.73		2.30	1.01-5.20	
McCabe/Jackson (%)			0.95			0.18
Non fatal	1.00	-	-	1.00	-	-
Fatal within 5 years	0.98	0.54-1.77	0.95	1.71	0.88-3.31	0.11
Fatal within 6 months	1.15	0.45-2.93	0.76	0.50	0.06-3.87	0.50
Gender			0.06*			0.001*
Male	1.00	-		1.00	-	
Female	1.61	0.98-2.65		3.27	1.65-6.48	
Hemiplegia			0.78			0.03*
No	1.00	-		1.00	-	
Yes	1.10	0.55-2.20		2.10	1.07-4.11	
Immune suppression°			0.67			0.45
No	1.00	-		1.00	-	
Yes	1.18	0.54-2.58		1.42	0.57-3.51	

*Independent variables significantly (or slightly significantly) associated with urinary tract infection.

*ASA* = American Society of Anesthesiologists; autoimmune disease, transplantation, hepatopathy, nephropathy, neoplasia.

**Table 3:** **Multivariate logistic regression models clustered on hospitals presenting independent factors associated with urinary tract infection stratified on gender, *Swiss-NOSO *Nationwide Prevalence Study, 2004**

**Variables**	**Male (n = 3703)**			**Female (n = 4171)**		
	**Odds ratio**	**95% CI**	**P value**	**Odds ratio**	**95% CI**	**P value**
Age groups (%)			0.01*			0.10*
<70 years	1.00	-		1.00	-	-
> = 71 years	2.57	1.24-5.31		1.51	0.93-2.45	
Recent surgical intervention			0.78			0.43
No	1.00	-		1.00	-	
Yes	1.11	0.55-2.21		0.82	0.49-1.35	
ASA score (%)			0.30			0.47
<4 pts	1.00	-		1.00	-	-
4-5 pts	0.52	0.15-1.81		0.73	0.31-1.73	
Charlson group			0.50			0.89
0-3 pts	1.00	-		1.00	-	
> = 4 pts	1.44	0.50-4.11		0.94	0.39-2.28	
Length of stay			0.57			<0.001*
<20 days	1.00	-	-	1.00	-	-
21-40 days	0.86	0.26-2.92	0.81	2.75	1.52-4.96	0.001
> = 41 days	1.75	0.59-5.17	0.31	2.89	1.41-5.92	0.004
Recent stay in intensive care			0.59			0.04*
No	1.00	-		1.00	-	
Yes	0.79	0.35-1.81		1.83	1.03-3.24	
McCabe classification (%)			0.84			0.14*
Non fatal	1.00	-	-	1.00	-	-
Fatal within 5 years	0.81	0.36-1.82	0.61	1.70	0.99-2.91	0.05
Fatal within 6 months	1.11	0.31-4.04	0.87	0.98	0.32-3.00	0.98
Prior exposure to urinary catheter			<0.001*			<0.001*
No	1.00	-		1.00	-	
Yes	6.77	3.20-14.35		3.04	1.83-5.04	
Hemiplegia			0.08*			0.34
No	1.00	-		1.00	-	
Yes	2.04	0.93-4.46		1.36	0.73-2.54	
Immune suppression°			0.39			0.57
No	1.00	-		1.00	-	
Yes	1.55	0.57-4.18		1.24	0.58-2.65	

*Independent variables significantly (or slightly significantly) associated with urinary tract infection.

*ASA* American Society of Anesthesiologists; autoimmune disease, transplantation, hepatopathy, nephropathy, neoplasia.

#### UTI with and without prior UC exposure

In the model assessing the likelihood of UTI stratified on prior exposure to UC (Table 2), the only factor significantly associated was a prolonged hospital stay > 40 days, while female gender showed only a tendency for association. In the model stratified on no prior exposure to UC, female gender, hemiplegia, and a recent stay in intensive care all increased significantly the odds for UTI.

#### UTI stratified by gender

In the model assessing the likelihood of UTI stratified by gender (Table 3), prior exposure to UC was significantly associated with UTI for both, whereas the length of hospital stay was significantly associated with UTI for women, but not for men.

#### Exposure to UC

We investigated the variables associated with the use of UC after stratification by gender. There were no differences between genders according to exposure to UC. The following factors were independently associated with a higher odds for the use of UC: age >70 years; recent surgical intervention; ASA score > 4 points; Charlson index > 4 points; high McCabe classification; recent stay in intensive care; and hemiplegia.

## Discussion

In this large study of patients hospitalised in Swiss acute care facilities, 25% were exposed to UC and 1.5% developed symptomatic UTI. Congruent with our results and other national and regional prevalence studies, a European report estimated a prevalence of nosocomial UTI of 1.65% (Table 4). Our findings mirror reports revealing a prevalence of UC use of 20.3% in emergency departments [8], 32% to 36% in acute care wards [5], and similar rates in most surveys conducted elsewhere (Table 4). The median duration of exposure to UC was also congruent with the literature, i.e., 2–4 days [18-41].

**Table 4:** Prevalence of urinary catheter use and/or symptomatic healthcare-associated urinary tract infection: reports in the peer-reviewed literature (January 1980-December 2012)

**Author/s**	**Population**	**Methodology**	**Catheter use**	**Infection**	**Remarks**
Jepsen et al. [19] 1982	40 hospitals in eight countries, n = 3899	Point prevalence	10.1% men, 11.8% women	6.5%	Conducted in 1980
Moro et al. [20] 1985	130 hospitals, n = 34,577	Point prevalence	9.4%	2.1%	National prevalence survey in Italy, 1983
Mertens et al. [21] 1987	106 hospitals, n = 8723	Point prevalence	15.7%	4.4%	National prevalence survey in Belgium 1984 70% surgery. Definition nosocomial: > 3rd day
Srámova et al. [22] 1988	23 hospitals, n = 12,260	Point prevalence		1.5%	Prevalence survey in Czechoslovakia, 1984
Emmerson et al. [23] 1996	157 centres, n = 37,111	Survey	-	2.4%	Prevalence survey in UK and Ireland, 1994
Gastmeier et al. [24] 1997	72 hospitals, n = 14,966	Point prevalence		1.1%	National prevalence survey in Germany, 1994
Scheel et al. [25] 1999	All acute care hospitals, n = 12,755	Point prevalence	-	2.2%	National prevalence survey in Norway, 1997
Vaqué et al. [26] 1999	n = 51,674 in 1997	Point prevalence	-	2.1%	National prevalence surveys in Spain, 1990-1997
French Prevalence Group [27] 2000	830 hospitals, n = 236,334	Point prevalence	9.6%	1.6%	National prevalence survey in France, 1996, including psychiatric and long-term care wards
Eriksen et al. [2] 2002	Acute care hospitals, n = 11,500-12,500	Point prevalence	-	1.7-2.0%	National prevalence surveys in Norway, 2002 and 2003
Gikas et al. [28] 2002	n = 3925	Point prevalence	8.6%	2.1%	Survey in 14 Greek hospitals, 1999
Lizioli et al. [29] 2003	Public hospitals, n = 18,667	Point prevalence	-	1.6%	Prevalence survey in Lombardy, 2000
Klavs et al. [30] 2003	Acute care, n = 6695	Point prevalence	-	1.2%	National prevalence survey in Slovenia, 2001
Nicastri et al. [31] 2003	15 hospitals in Italy, n = 2165	Point prevalence	22.4%	1.7%	All participating hospitals have > 400 beds
Wald et al. [5] 2005	Surgery, n = 111,330 523 Medicare hospitals	Retrospective cohort study	32% at discharge day	-	Patients at discharge after hip replacement
Tammelin [32] 2005	31 hospitals, n = 6369		16.5%	1.65%	Acute hospitals and long-term care facilities in Sweden, 2002
Gravel et al. [33] 2007	n = 5750	Point prevalence	22%	3.4%	National prevalence survey in Canada, 2002
Hopmans et al. [34] 2007	2 tertiary Dutch hospitals, n = 2661	Point prevalence twice a year	-	2.3% (1.2%-3.4%)	2001-2004. Obstetric wards excluded
Kevens et al. [35] 2007	445 US hospitals, n = 33,726,611	Throughout the year 2002	-	1.3%	Estimations for the USA
Pelizzer et al. [36] 2008	21 Italian hospitals, n = 6352	Period prevalence	25.2%	2.2%	Prevalence study in Veneto region, Italy 2003
van den Broek et al. [37] 2011	10 hospitals, n = 16,495	Period prevalence	20.2%	2.6%	Netherlands, acute care hospitals
Cairns et al. [38] 2011	45 acute care hospitals, n = 11,090	Point prevalence	20.3%	2.0%	Scotland 2006, exclusion of obstetric patients
Cotter et al. [39] 2012	69 long-term care facilities, n = 4,170	Point prevalence	5.6%	1.5%	Long-term care facilities in Ireland, June 2010
Askarian et al. [40] 2012	8 university hospitals, n = 3450	Point prevalence	23.1%	1.4%	University hospitals in Shiraz, Iran
Health Protection Agency [41] 2012	103 healthcare facilities, n = 52,443	Point prevalence	18.8%	1.1%	English national point prevalence survey preliminary data
*Present article*	Acute care hospitals, n = 8169	Period prevalence Cluster-adapted	24%	1.5%	National prevalence survey in Switzerland, 2004

* Only reports including at least 2000 patients admitted to acute care facilities are included.

By contrast, we are not able to compare healthcare-associated UTI prevalence with prevalence encountered in the community. Scientific data on the incidence or prevalence of UTI in the Swiss general population are non-existent, while the literature provides only data in predefined populations, such as elderly men or diabetic patients. Moreover, different survey studies define UTI differently, e.g., by excluding or including asymptomatic colonization. However, according to rough data, the overall life-long incidence of UTI could be around 2-4% for young males [42], 6.3% for older Scandinavian males [43], and up to 20% for females. Among Saudi diabetic males, the prevalence might be as high as 7%, whereas asymptomatic urinary tract colonization might be as high as 41% in diabetic women [44].

We found a significant association between UTI and UC use. However, the relation between prior exposure to UC and subsequent UTI was much less systematic than expected, and UC use preceded UTI in only 58% of cases. This does not appear to be unique to our study. In a European study reviewing 4.4 million admissions in 1999, 37.2% of all UTI episodes did not reveal prior exposure to UC [4]. A proportion of 41% UTI without prior exposure to UC has been similarly evidenced in Italian hospitals [31], while Jespen et al. found 56.7% of patients with healthcare-associated UTI attributed to prior UC use [19]. These reports did not further explore the relative low frequency of UC use prior to UTI.

In our study population, the low rate of UTI may reflect the low UC utilization rates. This is not surprising. One explanation might be recent surgery and/or a short ICU stay as these patients are often exposed to UC and acquire UTI more frequently than those hospitalised on medical wards. According to survey definitions, UC use was only recorded when it lasted > 24 h, but some patients undergoing surgery could have been exposed to UC for a shorter time, at least during surgery or ICU stay. Although it cannot be excluded that short duration catheterisation might predispose to subsequent UTI, this is unlikely given the low UTI incidence of only 2% [45] to 2.5% [46] at 24 h of UC use. Moreover, at least in the largest institution involved in the current study, this assumption would not be true [47]. Since 2001, UC during surgery is restricted to patients with a foreseen duration of more than 5 h or for arthroplasty surgery if the patient meets one of the following conditions: age >75 years; ASA class >3 points; presence of morbid obesity or urinary incontinence [43,48]. Thus, only 15.7% of all orthopaedic surgery is performed with a UC in place. For postanaesthesia care, only 4.7% of all patients required bladder catheterisation without permanent insertion of a UC [46].

Another population prone to intermittent catheterisation are patients with neurogenic bladders who were not identified as such in our study protocol [49,50]. Of note, our study involved acute care settings with the voluntary exclusion of long-term care facilities or homes where most patients with neurogenic bladders are usually housed. Thus, the prevalence of neurogenic bladders in acute care settings should be low. We consider that we have avoided a major bias by including paraplegic patients in the study population. Finally, the exact risk of symptomatic UTI after one single intermittent catheterisation is unknown. Most of the literature on intermittent catheterisation concerns individuals with repeated catheterisation due to neurologic problems [49,50]. Among these patients with intermittent self-catheterisation during several years or months, the cumulative risk of UTI is reported to be as high as 50% according to several surveys [50]. However, these patients cannot be compared with individuals with normal bladders who are only undergoing one single intermittent catheterisation for anaesthesiological or surgical reasons.

UTI can complicate urological interventions and be technically classified as surgical site infection. In a literature review, Slade reported approximately 19% UTI after urological surgery [48]. We are unable to provide information on the proportion of “official urological patients” among all those undergoing surgery, because many Swiss surgical wards are mixed, especially in smaller hospitals, are mixed and care for urology and non-urology patients at the same time. Similarly, the same surgeons may often perform urologic and other surgical interventions during the same day. However, it is unlikely that these urologic patients represent a large group and that freshly-operated urological patients would have been exposed to UC for more than 24 h. Specialized, urology-only, surgical wards did not exist in Switzerland at the time of the study and urology patients constitute a maximum of 10% of all surgical patients in many university centres. As summarised in Table 4, few national prevalence studies further stratify or report surgical specialties in detail. In the studies by Emmerson et al. [23] and Sramova et al. [22], the proportion of patients hospitalised on urological wards was only 3.9% and 2.4%, respectively.

Our survey has several limitations. i) The study design was not targeted towards delineating the origin of UTI. The causal inference between exposure to UC and UTI seems logical, but is not proven *sensu strictu*. ii) Despite the large number of patients included, only 126 acquired UTI. Although positive in terms of infection control, these small numbers are associated with reduced statistical power that is recognized in the wide confidence intervals. iii) Results are limited to acute care sectors. Many patients exposed to UC in high-income countries live in nursing homes or other long-term care facilities where the prevalence is higher and UTI is one of the most frequent infections [1,10]. iv) Data related to antibiotic administration or urine acidification are lacking. This could be important as patients treated for other infections might be protected from UTI with antimicrobials covering Gram-negative rods, while antibiotic administration prior to hospital admission might equally have diminished the bacterial burden in the genitourinary system. To the best of our knowledge, no prevalence study has explored this theoretical relationship. Of note, in our study, symptomatic UTI occurred on average two weeks after hospital admission, but we ignore if patients already had asymptomatic urinary tract colonization before hospital admission. v) So far, only prevalence studies and personal clinical experience report a high proportion of UTI without prior exposure to UC. There are no prospective cohort studies or randomized trials to confirm this ubiquitous finding. We currently ignore if the ability to track the catheter as a risk factor for nosocomial UTI might be limited when using prevalence studies. vi) Our study protocol did not target differences in the clinical presentation between UTI with and without prior UC use. This may be a bias as the presence of UC may influence physicians in the work-up of fever, i.e., they might order urine cultures more often in patients with UC. This theoretical bias is likely to shift the proportion of UTI towards UC use.

In conclusion, exposure to UC is the most important risk factor for healthcare-associated UTI according to prevalence studies, but only for a proportion of all patients ── at best two-thirds. This finding appears to be shared by other local or national prevalence studies. In our study, the separate analysis for UTI in the absence of prior UC use revealed only female gender [48], hemiplegia [5], and prolonged hospital stay as significant risk factors. The cumulative impact of other less inalienable risk factors should not be underestimated. Further research needs to place an emphasis on innovative strategies to address the specific issues of UTI in the absence of exposure to UC.

## Competing interests

The authors declare that they have no competing interests.

## Authors’ contributions

The national prevalence study was designed by all authors. The study was primarily coordinated by HS and IU with support from all authors, including identifying patients and collecting data on cases. IU, HS and AG managed the data and performed the initial analysis of cases. IU, AG, and DP drafted the manuscript. All authors interpreted the results and revised the manuscript.

## Financial support

This study was supported by the Swiss Federal Office for Public Health and unrestricted educational grants from Astra Zeneca, Bayer (Switzerland) AG, BBraun Medical AG, Beiersdorf AG, Ecolab GmBH, Mundipharma AG, and Schülke + Mayr AG.
